# Synthesis of Selenium Nanoparticles Modified by Quaternary Chitosan Covalently Bonded with Gallic Acid

**DOI:** 10.3390/polym15092123

**Published:** 2023-04-29

**Authors:** Alexey Lunkov, Mariya Konovalova, Balzhima Shagdarova, Yuliya Zhuikova, Alla Il’ina, Valery Varlamov

**Affiliations:** 1 Institute of Bioengineering, Research Center of Biotechnology, Russian Academy of Sciences, Moscow 119071, Russia; zhuikova.uv@gmail.com (Y.Z.); ilyina@biengi.ac.ru (A.I.); varlamov@biengi.ac.ru (V.V.); 2 Shemyakin-Ovchinnikov Institute of Bioorganic Chemistry, Russian Academy of Sciences, Moscow 117997, Russia; mariya.v.konovalova@gmail.com

**Keywords:** chitosan, quaternary chitosan, selenium nanoparticles, cytotoxicity

## Abstract

Quaternary chitosan derivative with covalently bonded antioxidant (QCG) was used as media for synthesis of selenium nanoparticles (SeNPs). SeNPs were characterized using AFM, TEM, and DLS methods. The data confirmed the formation of stable nanoparticles with a positive charge (34.86–46.73 mV) and a size in the range 119.5–238.6 nm. The antibacterial and fungicidal activity of SeNPs occurred within the range of values for chitosan derivatives. In all cases, the highest activity was against *C. albicans* (MIC 125 µg/mL). The toxicity of the modified selenium nanoparticles to eukaryotic cells was significantly higher. Among nanoparticle samples, SeNPs that were synthesized at 55 °C demonstrated the highest toxicity against Colo357 and HaCaT cell lines. Based on these results, SeNPs loaded with doxorubicin were obtained. DOX loading efficiency was about 18%. QCG-SeNPs loaded with DOX at a concentration of 1.25 μg/mL inhibited more than 50% of hepatocarcinoma (Colo 357) cells and about 70% of keratinocytes (HaCaT).

## 1. Introduction

Selenium (Se) is an important micronutrient for many living organisms [1]; its chemical properties are very similar to sulfur [2]. However, unlike sulfur, the chemical transformations of selenium in “living systems” are characterized primarily by the simple reversibility—“easy in—easy out” [3]. It is known today that selenoproteins have various functions, such as homeostasis of hormones, protection against damage by proteins and lipids, antioxidant and anti-inflammatory functions, regulation of cell proliferation and apoptosis, and protein folding [4]. Se plays the key role as redox center of various antioxidant enzymes such as glutathione peroxidase (GPX), thioredoxin reductase (TXNRD), and selenoprotein P (SELENOP) [5]. Se compounds obtained in the diet are rapidly metabolized via the trans-selenation pathway or reduced in the presence of glutathione (GSH) for the production of the common intermediate, selenocysteine [6], an amino acid that is incorporated into peptide chains of selenoproteins. The first evidence of Se involvement in cardiovascular function came from the discovery that Se deficiency was involved in Keshan’s disease (KD), a severe form of cardiomyopathy that is sometimes fatal [7]. However, the role of Se in cardiovascular syndromes, particularly under dietary Se deficiency, remains only partially understood. The relationship between Se status and cancer has been debated for a long time and the results of studies are contradictory [8]. Some researchers have suggested that the presence of Se in food protects against cancer [9]. Se nanoparticles (SeNPs) have attracted significant attention from researchers due to their unique biological, catalytic, photoelectric, and semiconductor properties. SeNPs are potentially used in biomedicine to treat many diseases, including cancer, diabetes, inflammatory diseases, liver fibrosis, and drug-induced nephrotoxicity [5,10]. The synthesis and application of SeNPs attracted attention due to several advantages, including chemical stability, biocompatibility, and low toxicity [11]. Recently, the aminopolysaccharide chitosan and its derivatives have been widely regarded as stabilizing and surface-modifying agents in the synthesis of nanoparticles, including SeNPs [12]. Chemical modification of chitosan with the introduction of additional functional groups and biologically active substances opens up great opportunities for changing the final properties of nanomaterials [13]. Previously, researchers [14] reported a difference in nanoparticle release when a different MW chitosan was used to stabilize SeNPs. Researchers [15] have shown the influence of the structure of chitosan derivatives (containing covalently bonded gallic and folic acids) on the shape of nanoparticles. Modification of nanoparticles using trimethyl chitosan and doxorubicin was considered in a study [16].

The aim of our work was the synthesis and characterization of SeNPs stabilized by quaternized chitosan derivatives and the study of their biological properties. In our study, low molecular weight chitosan was used as a starting material. It was further modified using glycidyltrimethylammonium chloride (GTMAC) and the introduction of gallic acid using activated esters into the structure. Previously, this water-soluble derivative was considered the basis for the synthesis of AgNPs. Quaternary ammonium (CH_3_)_3_-N^+^-CH_2_-CHOH-CH_2_- provides solubility in a wide pH range and the presence of a phenolic fragment—affinity to proteins, thus reducing activity. In addition, a high positive charge ensures the stability of nanoparticles in an aqueous medium and the possibility of additional ionic interaction with a negatively charged cell membrane. 

## 2. Materials and Methods

### 2.1. Materials and Reagents

Chitosan from crab shells (Bioprogress LLC, Shchelkovo, Russian Federation; MW = 1040 kDa, DD = 85%) was hydrolyzed using nitric acid to obtain a fraction with MW = 28 kDa, DD = 93% [17], on the basis of which all derivatives were synthesized. Chitosan was modified using the GTMAC reagent and gallic acid (Sigma-Aldrich, St. Louis, MO, USA). The Folin–Ciocalteu reagent (Merck, New York, NY, USA) was used to determine the amount of gallic acid in the final compound. Chitosan and chitosan derivatives were characterized by 1H-NMR. Samples were prepared in acidified deuterated water. The proton spectrum was recorded on a Bruker AMX 400 spectrometer (Bruker, Billerica, MA, USA); 4,4-dimethyl-4-silapentane-sulfonic acid was used as a standard. Selenium oxide (IV) and L-ascorbic acid (Fisher Scientific, Hampton, TN, USA) were used to synthesize SeNPs. We use dialysis tubing, MWCO 3.5 kDa from regenerated cellulose, RC, diameter 16 mm (SERVA Electrophoresis GmbH, Heidelberg, Germany) to remove byproducts. Tetrazolium hydrochloride was used in the MTT test (Sigma-Aldrich, St. Louis, MO, USA); Roswell Park Memorial Institute medium (RPMI) and Dulbecco’s modified Eagle’s medium (DMEM) containing 7 % of fetal bovine serum, glutamine, and penicillin/streptomycin (PanEco, Moscow, Russian Federation). Cells were trypsinized using a trypsin/EDTA solution (PanEco, Moscow, Russian Federation). Other reagents and solvents used in the study were of analytical grade. UV spectra were recorded on a UV 1601 instrument (Schimadzu Corp, Kyoto, Japan). TEM images and EDX spectra of SeNPs were obtained using a JEM 1400 electron microscope (JEOL Ltd., Tokyo, Japan) equipped with an INCA Energy TEM 350 EDS system (Oxford Instruments plc, Abingdon, UK); DLS analysis of nanoparticle samples was performed using Zeta Plus particle analyzer (Brookhaven Instruments Corp, New York, USA); AFM studies were performed on an INTEGRA Prima atomic force microscope (NT-MDT SI, Moscow, Russian Federation). GloMax-Multi Detection System (Promega Corp, Madison, WI, USA) was used for the determination of doxorubicin quantity. Multiskan FC (ThermoFisher Scientific Inc, Waltham, WI, USA) plate reader was used in MTT tests. 

### 2.2. Synthesis of Quaternary Chitosan with Gallic Acid (QCG)

The synthesis of quaternary chitosan (QC) was carried out according to the described method [18]. The introduction of gallic acid was based on a modified procedure [19]. Solution I: 1g of QC in 20 mL of distilled water acidified with 0.1M HCl to pH = 4.5. Solution II: EDC (0.594 g; 3.1 mmol), NHS (0.357 g; 3.1 mmol) were mixed as solid, added to 10 mL of gallic acid (0.528 g; 3.12 mmol) in water/acetone (1/1). Solution II was placed on the ice bath. After 1h, Solution II was gradually added to Solution I. The resulting reaction mixture was placed in an ice bath for 1 h. After 24 h at RT, the product was precipitated with acetone. The precipitate was placed on filter and washed with ethyl alcohol (3 × 50 mL). The final product was dialyzed against acidified water at pH = 5.0 for 24 h and freeze-dried.

### 2.3. DPPH Inhibitory Activity of QCG

The antioxidant activity of the chitosan derivative was characterized by the inhibition of the stable DPPH radical in according to the procedure [20]. The concentration of the tested substances was 0, 50, 100, 250, 500, and 1000 μg/mL. Ascorbic acid was used as a comparison antioxidant. Optical density was measured at a wavelength of 517 nm. The ability to absorb radicals was calculated using the following ratio (1):

(1) $$Scavenging\;activity\left(\%\right)=\left(1-\frac{A_{sample}}{A_{control}}\right)\times 100\%$$

A_sample_, A_control_—optical density of sample and control solution.

### 2.4. Synthesis of Selenium Nanoparticles in the Presence of QCG 

SeNPs stabilized by quaternized chitosan were obtained as a result of the reduction of Se^+4^ in the presence of a chitosan derivative. First, 10 mL of a QCG solution (1 mg/mL) in 0.5% acetic acid was mixed with 10 mL of an H_2_SeO_3_ solution (10 mM) (60 µL of DOX solution (2 mM) was also mixed in to obtain QCG-SeNPs + DOX). After 10 min, 10 mL of L-ascorbic acid solution (30 mM) was added dropwise to the resulting reaction mass. The synthesis was carried out at different temperatures: 25, 55, and 80 °C for 1 h. During the reduction of selenium, a color change of the reaction mixture was observed from colorless to orange-red. SeNPs were then centrifuged at 14,000 rcf, redispersed in distilled water, and dialyzed against distilled water (MWCO 3.5 kDa) for 6 h.

### 2.5. DPPH Inhibition Activity of QCG

The antioxidant properties of QCG-SeNPs were characterized by inhibition of the stable DPPH radical in accordance with above-described procedure in 2.3. First, UV spectra were obtained in order to compare the NPs spectra to the DPPH spectra. Nanoparticle solutions were additionally centrifugated to prevent light scattering during measurement of absorbance.

### 2.6. Characterization of QCG-SeNPs

The size and shape of selenium nanoparticles were characterized by transmission electron microscopy using a JEM 1400 electron microscope. Elemental analysis was performed using the INCA Energy TEM 350 EDS system. The particle size distribution, average size, and zeta potential of the nanodispersed system were determined using a Zeta Plus particle analyzer, based on dynamic light scattering (DLS) using built-in software to analyze the measurement results. The dimensional characteristics of the nanoparticles were obtained by atomic force microscopy (AFM) in semi-contact mode in air using the Image Analysis program (NT-MDT SI, Moscow, Russian Federation). From 5 to 10 images with a size of 10 × 10 µm for each sample were obtained and analyzed. The DOX content of the QCG-SeNPs DOX samples was determined by fluorescence spectroscopy. The reaction mass was centrifuged at 14,000 rcf, then the fluorescence of the supernatant was measured, and this amount was subtracted from the load mass to obtain the amount of DOX in the nanoparticles. The following ratio (2) was used to calculate the loading of nanoparticles:

(2) $$DOX\;Loading\;efficiency\left(\%\right)=(\frac{m\left(DOX\;in\;QCG-SeNPs\right)}{m\left(DOX\;total\;amount\;added\right)})\times 100$$

### 2.7. Study of the Antibacterial and Fungicidal Properties of QCG-SeNPs

To determine the antibacterial activity, we used strains of *E. coli* ATCC 25922, *S. epidermidis* 33 GISK, and *S. aureus* ATCC 35591. Bacterial cultures were stored in LB-agar (LB Agar Miller) at 4 °C. To prepare the inoculum or suspension, a single colony of bacteria was transferred into 20 mL of LB (LB Broth Miller) medium and incubated on a shaker at 150 rpm at 37 °C for 18 h. The minimum inhibitory concentration (MIC) of the NPs was determined by the method of serial dilutions in a liquid medium according to the method [18]. 

To determine the fungicidal activity, we used the strain *C. albicans* ATCC 90028. The isolated yeasts were grown and stored on Sabouraud dextrose agar at 4 °C. To prepare the inoculum or suspension, a single colony of yeast cells was transferred into 20 mL of Sabouraud glucose broth and shaken for 24 h at 150 rpm. The MIC of the NPs was defined as the lowest concentration required to suppress cell multiplication by the method of serial dilutions in a liquid medium according to the method [21].

### 2.8. Study of the Cytotoxic Properties of QCG-SeNPs

Cytotoxicity of SeNPs was investigated by MTT assay [22,23] using the HaCaT and Colo357 cell lines. Cells were cultured in RPMI or DMEM. Cells were trypsinized using a trypsin/EDTA solution. The solutions containing nanoparticles were diluted to reduce their concentration from 100 µg/mL to 3.125 µg/mL. The cells were seeded at 10,000 cell/well and incubated for 72 h in a CO_2_-incubator at 37 °C. MTT was added to each well for the last 4 h. The culture medium was eliminated from the wells, and formazan crystals were dissolved in 100 μL of DMSO for 20 min. The optical density was measured at 540 nm using a plate reader, and cytotoxicity indices were calculated as follows, Equation (3):

(3) $$Inhibition\;Index=1-\frac{D_{540\;sample}}{D_{540\;control}}$$

where D_540 sample_ and D_540 control_ are the optical densities of the sample and control, respectively.

## 3. Results and Discussion

### 3.1. Synthesis of Quaternized Chitosan with Gallic Acid (QCG) and Its Characteristics

**Figure 1:**
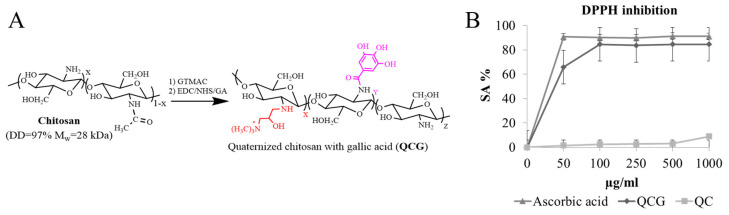
(**A**)—QCG synthesis scheme; (**B**)—Inhibition of model DPPH radicals by QCG derivative.

Low molecular weight chitosan (MW 28 kDa, DD 93%) was chemically modified. The derivative had a quaternary fragment (N-(2-hydroxyl)propyl-3-trimethyl ammonium) and covalently bonded gallic acid. The reaction scheme is presented in Figure 1A.

The synthesis of quaternized chitosan was carried out at 85 °C, and the chemical reaction was performed in a heterogeneous medium with a pH 6.5. It is known that under neutral and acidic conditions of the reaction medium, nucleophilic substitution takes place mainly along the amino group [24]. Gallic acid and its derivatives not only exhibit excellent antioxidant, anticarcinogenic, antimutagenic, and antimicrobial properties but also protect cells against oxidative stress [25]. The antioxidant activity of quaternized chitosan was increased by the grafting of gallic acid to its amino groups in the presence of 1-ethyl-3-(3-dimethylaminopropyl)-carbodiimide (EDC)/N-hydroxysuccinimide (NHS). The structure was proven using NMR H^1^ and FTIR spectroscopy in the previous work [26]. The degree of substitution (53%) was determined by conductometric titration with AgNO_3_. Gallic acid content (273 mg/mL) was determined using the Folin–Ciocalteu reagent. QCG demonstrated a significant increase in DPPH inhibition activity in comparison to QC in the concentration range of 50-1000 µg/mL. QCG activity was comparable to that of ascorbic acid (Figure 1B). 

### 3.2. Synthesis of Selenium Nanoparticles in the Presence of QCG

**Figure 2:**
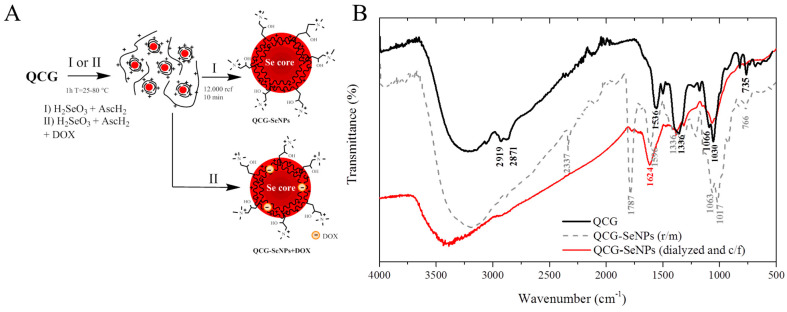
(**A**)—Synthesis scheme and appearance of selenium nanoparticles stabilized by quaternized chitosan with gallic acid QCG−SeNPs; (**B**)—IR spectroscopy data: chitosan derivative (QCG); reaction mass with NPs, (QCG−SeNPs r/m) and stabilized NPs (QCG−SeNPs dialyzed and c/f).

QCG was used as a stabilizing agent in the synthesis of nanoparticles. Ascorbic acid was used as a reducing agent. The synthesis scheme is shown in Figure 2A. The interaction of QCG with SeNPs was studied using FTIR spectroscopy (Figure 2B). 

In the structure of the initial derivative, typical OH polysaccharide vibrations in the region of 3200 cm^−1^, NH vibrations at 1536 cm^−1^, C-N vibrations at 1336 cm^−1^, and C-H 1066, 1030 cm^−1^ were observed. In the reaction mass of QCG-SeNPs, a peak appears at 1787 cm^−1^, 1611 cm^−1^ corresponding to C=O and C-O vibrations in the structure of ascorbic acid, respectively, and the peaks of C-N and C-H vibrations were preserved. After dialysis and centrifugation, the peak responsible for ascorbic acid disappeared; the original peaks of the modified polysaccharide remained, however, they became less intense, and a shift in NH vibrations was observed, which may indicate the participation of the amino group in the stabilization of SeNPs. The modified polymer provided hydrophilic–hydrophobic interactions at the surface of nanoparticles. SeNPs were obtained in an aqueous solution without a stabilizer (as a control) and in the presence of QCG in the temperature range from 25 to 80 °C. An increase in the color intensity of the solution was observed with an increase in the reaction temperature (Figure 2A). The formation of nanoparticles had already occurred at room temperature for several minutes. The particles obtained in solutions in the presence of QCG had higher stability compared to the control. In the control, sedimentation and aggregation of particles were observed within 2 h. The UV spectrum of the reaction mixture showed an increase in intensity and a shift in the absorption peak from 259 to 262–293 nm due to the contribution of surface plasmon resonance (SPR) and QCG in the formed nanoparticles (Table 1). It was previously suggested by researchers that gallic acid covalently bound to chitosan provided the presence of rigid fragments of the polymer chain due to π-π stacking of adjacent polyphenolic substituents [15]. The presence of a hydrophilic positively charged ammonium group provides the solubility of the polymer and the stabilization of nanoparticles in solution. The formation of an electric double layer (DES) on the surface of nanoparticles prevents their aggregation.

### 3.3. DPPH Inhibition of QCG-SeNPs

**Figure 3:**
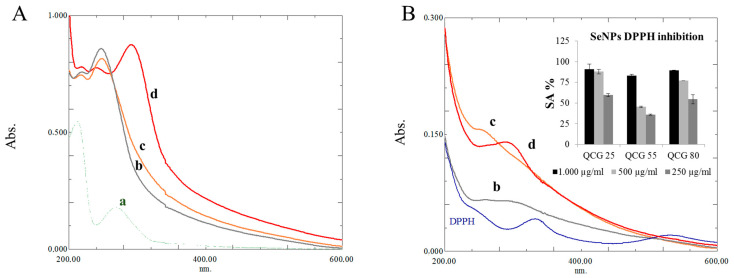
(**A**)—UV-spectra: a—QCG derivative; b,c,d—QCG-SeNPs-25, 55, 80, respectively; (**B**)—DPPH inhibition activity of QCG-SeNPs, respectively by spectrophotometry (%) and UV-spectroscopy data (b,c,d—QCG-SeNPs-25, 55, 80) for inhibition of DPPH.

To obtain the QCG chitosan derivative, according to [27] we used a low molecular weight 28 kDa chitosan, since the quaternary chitosan 40 kDa had stronger antioxidant effect than 160 kDa quaternary chitosan. Many researchers conclude about reducing power and antioxidant activity of SeNPs [28]. Visual changes and the UV spectra of the obtained solutions clearly showed the reduction in DPPH radicals. Reducing power of SeNPs was comparable with QCG derivative of chitosan. Thus, the reducing power is mainly attributed to the proton donating activity of gallic acid on the surface of SeNPs. From the results of the DPPH experiment, a decrease in particle size leads to an increase in specific surface area. This increases the probability of contact between the heterogeneous phase and the radical in the homogeneous phase, i.e., in solution. The UV spectra of NPs and QCG derivative are presented in Figure 3A(a–d). 

In the concentration range from 250 to 1000 µg/mL, the highest activity was achieved by the QCG-SeNPs 25 sample, showing 59.55–90.73%. QCG-SeNPs 55 showed the lowest activity among SeNPs samples 35.89–83.04%. QCG-SeNPs 80 showed activity in the range 54.57–89.33%. The highest intensity of QCG-SeNPs 80 pike on UV spectra (Figure 3B(d)) indicates increasing number of SeNPs in the sample. Based on UV spectroscopy data for QCG-SeNPs, we can conclude that an increasing number of particles influence DPPH inhibition activity results, but as previously mentioned, the main activity is due to the presence of gallic acid.

### 3.4. Characterization of QCG-SeNPs by DLS, AFM, and TEM

**Figure 4:**
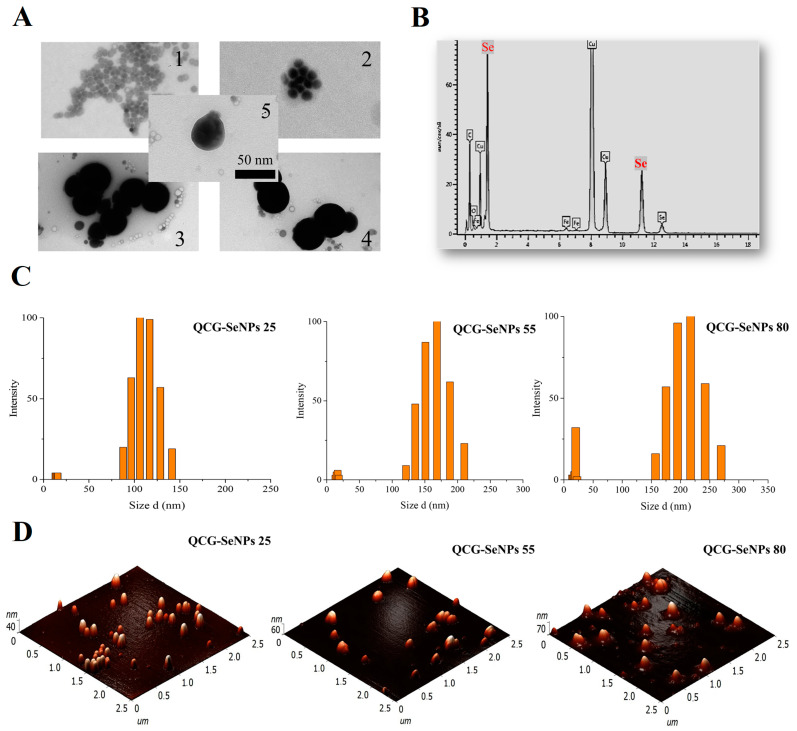
(**A**)—TEM image of selenium nanoparticles: 1—QCG-SeNPs 25; 2,5—QCG-SeNPs 55; 3,4—QCG-SeNPs 80; (**B**)—EDS elemental analysis results for QCG-SeNPs 25; (**C**)—histograms of particle size distribution obtained by the DLS method; (**D**)—AFM images of QCG-SeNPs.

Selenium nanoparticles were characterized by UV spectroscopy, DLS, AFM, and TEM. The results are presented in Table 1. Previously, researchers [15] stated the possibility of obtaining cubic selenium nanoparticles using trimethylchitosan derivatives with covalently bound gallic acid; however, according to TEM data, spherical selenium nanoparticles were formed in all cases. It may be related to large N-(2-hydroxyl)propyl-3-trimethyl ammonium fragments in macromolecular structure that influence the interaction of chitosan derivatives. In the case of QCG-SeNPs 25, low-density selenium particles were observed (Figure 4A(1)).

An increase in the density of SeNPs with increasing temperature was observed—in the case of QCG-SeNPs 55, aggregates of denser particles on Figure 4A(2) and individual particles on Figure 4A(5) were observed. A similar picture was observed for QCG-SeNPs 80; however, the formation of large aggregates was also observed in the samples on Figure 4A(3,4). The average size of the formed particles in all cases was about 50 nm. The presence of selenium was confirmed by the EDS analysis. Similar spectra were obtained. The results for QCG-SeNPs 55 are shown on Figure 4B. The results of studying the dimensional characteristics by the AFM method are presented in Table 1 and on Figure 4D. For a detailed study of the dimensional characteristics of nanoparticles, the method of dynamic light scattering was used. According to the DLS results, increasing the synthesis temperature caused an increase in particle size. Particle size distribution histograms are shown in Figure 4C. Furthermore, the particles obtained at a higher temperature had a higher value of the ζ-potential (Table 1). The high positive surface charge of SeNPs indicates the participation of quaternized chitosan in the stabilization of the formed nanostructure. The positive ζ-potential of the QCG-SeNPs sample corresponded to previous results for quaternized chitosan derivatives [18]. Among the nanoparticle samples, the temperature conditions of the sample with the highest toxicity in the MTT test were used to obtain selenium nanoparticles with doxorubicin. According to fluorometry, the loading of QCG-SeNPs with doxorubicin was 18%. According to the results of measuring the ζ-potential, a decrease in the positive charge of the particle surface from +45.76 to +37.00 mV was shown, most likely due to the incorporation of DOX into nanoparticles.

### 3.5. Antibacterial and Fungicidal Properties of QCG-SeNPs 

Nanoparticles stabilized by QCG demonstrated relatively low antibacterial activity. Apparently, the antibacterial effect of the nanomaterial is associated mainly with the residues of the positively charged polymer on the particles surface. This effect is due to the interaction of the positively charged quaternized chitosan derivative with the negatively charged cell wall [18]. The increase in activity tended to shift toward the smallest particle size of QCG-SeNPs 25 for *S. epidermidis*. This is probably due to the fact that small particles are more tightly in contact with the cell wall of the microorganism [29]. In all cases, the particles showed the lowest MIC of 125 μg/mL against *C. albicans*, which may be due to the interaction of Se with cellular proteins, in which Se displaces sulfur for sulfur-containing amino acids such as cysteine (Cys) and methionine (Met) [30,31]. Values obtained for *C. albicans* are consistent with literature data (70 μg/mL) [32]. Results are presented in Table 2.

### 3.6. Cytotoxicity of QCG-SeNPs

**Figure 5:**
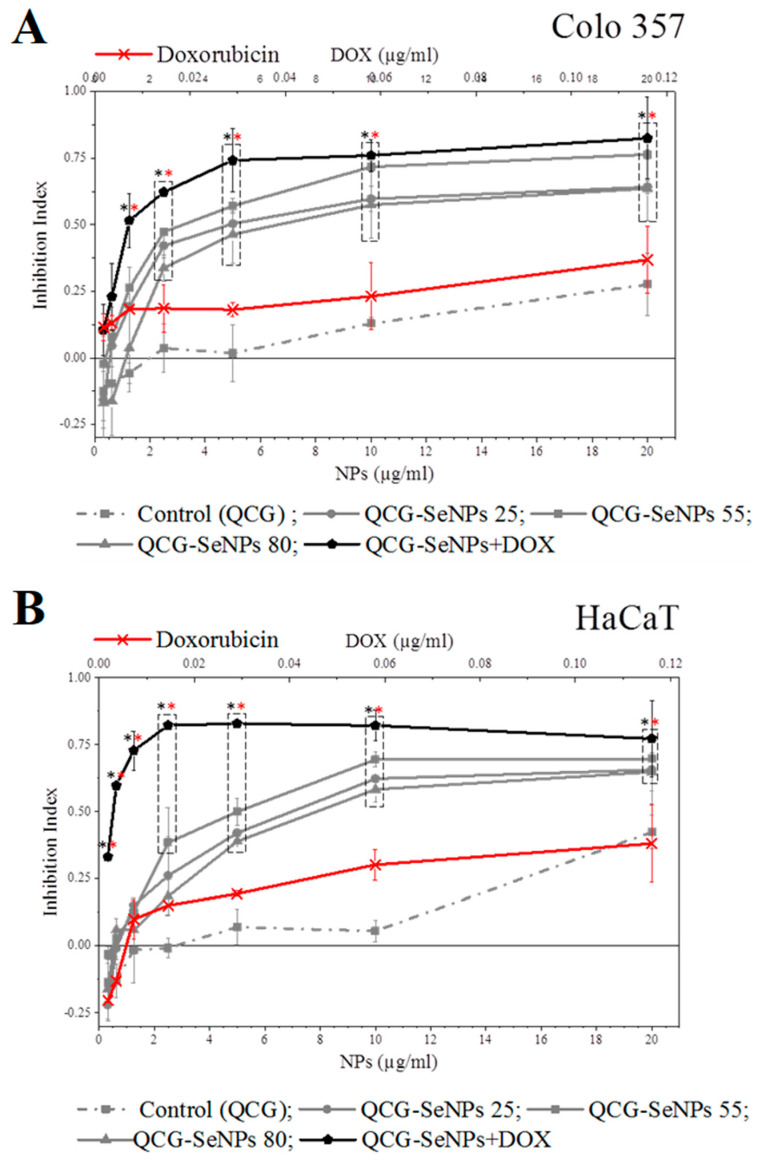
Cytotoxic effect of nanoparticles on HaCaT keratinocyte (**B**) and Colo357 hepatocarcinoma cell lines (**A**). The statistical significance was evaluated using the Student’s *t*-test: black stars—*p* < 0.05 vs. Control (QCG), red stars—*p* < 0.05 vs. DOX. Dotted rectangles indicate the points on the curves that are statistically different from the control or the DOX at that concentration.

The cytotoxic properties of the nanoparticles were examined on the cell lines of hepatocellular carcinoma Colo 357 and keratinocytes HaCaT. The cytotoxicity of the control—modified chitosan QCG was within 40% for the entire range of the studied concentrations on both types of cells (Figure 5). 

In both cases, the nanoparticles synthesized at 55 °C were more cytotoxic than the QCG-SeNPs 25, 80 samples. These particles inhibit the growth of HaCaT cells by 40–50%; similar data were obtained for SeNPs [33]. The QCG-SeNPs 55 inhibited the growth of Colo357 cells by 50–70%. The appropriate dimensional characteristics (162.6 nm) and the highest surface charge of the nanomaterial (+46.73 mV) provided the best penetration into the cell. One of the possible mechanisms of action of the chitosan derivative used in the particles on the cells is its positive charge, which adheres to the negatively charged surface of the cell membrane. This leads to difficulties in intercellular interaction and inhibition of proliferation. Since QCG-SeNPs 55 had the highest positive charge, it had the highest cytotoxic effect [34]. The conditions for synthesis of this sample were selected based on these parameters in order to obtain nanoparticles loaded with doxorubicin QCG-SeNPs 55 + DOX. As a control, we took the amount of DOX equivalent to the concentration in the reaction mass and diluted it similarly to NPs (Figure 5—x-axis (DOX µg/mL) from above). The cytotoxic effect of QCG-SeNPs + DOX was higher than that of doxorubicin and QCG-SeNPs 55. QCG-SeNPs + DOX at a concentration of 1.25 μg/mL inhibited more than 50% of cells, which is eight times higher than the value of QCG-SeNPs 55 at a similar concentration for Colo 357. At a concentration of 20 µg/mL, 84% of the cells were inhibited (Figure 5). A similar pattern was seen with HaCaT: doxorubicin administration significantly increased the toxicity of the QCG-SeNPs + DOX sample. QCG-SeNPs + DOX at a concentration of 1.25 μg/mL inhibited more than 70% of HaCaT cells. At a maximum concentration of 20 µg/mL, it inhibited 77% of the cells. The results are consistent with the literature data obtained for GA-modified selenium nanoparticles against HepG2 cells [35].

## 4. Conclusions

Water-soluble quaternized derivatives of low molecular weight chitosan can be used as a stabilizing agent in a simple and accessible method for the synthesis of selenium nanoparticles. Stable particles with a positive charge are formed. In the DPPH test, the obtained SeNPs demonstrated inhibition activity. The antibacterial and fungicidal activity of SeNPs occurred within the range of values for chitosan derivatives. In all cases, the greatest activity was in relation to *C. albicans*. The obtained modified selenium nanoparticles had a significantly higher toxicity towards eukaryotic cells; among them, particles of QCG-SeNPs 55 with an average diameter of 162.6 nm (DLS) and a zeta potential of 46.73 mV had the highest toxicity towards both hepatocarcinoma cell lines (Colo 357) and keratinocytes (HaCaT). Additional modification with the introduction of DOX into the stabilized selenium particles made it possible to increase their toxicity in comparison with unmodified particles. Further investigations will focus on incorporation of ligands to increase the selective toxicity of nanoparticles.

## Figures and Tables

**Table 1:** Characterization QCG-SeNPs, synthesized at different temperatures.

Sample	d (nm) DLS	d (nm) AFM	ζ-Potential (mV)	UV—Spectra λmax (nm)
QCG-SeNPs 25	119.5 ± 29.54	26.39 ± 8.55	+34.86 ± 2.24	246.0
QCG-SeNPs 55	162.6 ± 15.09	38.07 ± 10.05	+46.73 ± 2.21	247.5
QCG-SeNPs 80	238.6 ± 57.56	35.79 ± 16.42	+45.76 ± 2.33	293.0
QCG-SeNPs 55 + DOX	145.4 ± 28.14	-//-	+37.00 ± 2.24	-//-

**Table 2:** Antibacterial and fungicidal properties of QCG-SeNPs.

Sample	MIC (µg/mL)			
	*C. albicans*	*S. epidermidis*	*S. aureus*	*E. coli*
QCG-SeNPs 25	125	125	500	500
QCG-SeNPs 55	125	500	500	500
QCG-SeNPs 80	125	500	500	1000

## Data Availability

Not applicable.
